# Genomic Characterization by Whole-Exome Sequencing of Hypermobility Spectrum Disorder

**DOI:** 10.3390/genes13071269

**Published:** 2022-07-18

**Authors:** Gerardo J. Alanis-Funes, Saúl Lira-Albarrán, Jesús Hernández-Pérez, Mario A. Garza-Elizondo, Rocío Ortíz-López, César V. Elizondo, Augusto Rojas-Martinez, Rocío A. Chávez-Santoscoy, Claudia Rangel-Escareño

**Affiliations:** 1 School of Engineering and Sciences, Tecnologico de Monterrey, Campus Monterrey Av. Eugenio Garza Sada 2501 Sur, Monterrey 64849, N.L., Mexico; gerardo.alanisf@tec.mx (G.J.A.-F.); jhz.perez@tec.mx (J.H.-P.); 2 The Institute for Obesity Research, Tecnologico de Monterrey, Av. Eugenio Garza Sada 2501 Sur, Monterrey 64849, N.L., Mexico; saul.lira@tec.mx (S.L.-A.); rortizl@tec.mx (R.O.-L.); augusto.rojasmtz@tec.mx (A.R.-M.); 3 Instituto de Medicina Interna, Centro Médico Zambrano Hellion, Sistema Tec Salud, Monterrey 66278, N.L., Mexico; dr.mariogarza@medicos.tecsalud.mx (M.A.G.-E.); elizondovidal@gmail.com (C.V.E.); 4 School of Medicine and Health Sciences, Tecnologico de Monterrey, Monterrey 64710, N.L., Mexico; 5 School of Engineering and Sciences, Tecnologico de Monterrey, Campus Querétaro, Querétaro 76130, QRO., Mexico; 6 National Institute of Genomic Medicine, Mexico City 14610, CDMX, Mexico

**Keywords:** joint hypermobility, exome, genes

## Abstract

No genetic basis is currently established that differentiates hypermobility spectrum disorders (HSD) from hypermobile Ehlers–Danlos syndrome (hEDS). Diagnosis is entirely based on clinical parameters with high overlap, leading to frequent misdiagnosis of these two phenotypes. This study presents a landscape of DNA mutations through whole-exome sequencing of patients clinically diagnosed with generalized HSD. In this study, three genes (*MUC3A*, *RHBG*, and *ZNF717*) were mutated in all five patients evaluated. The functional enrichment analysis on all 1162 mutated genes identified the extracellular matrix (ECM) structural constituent as the primary overrepresented molecular function. Ingenuity pathway analysis identified relevant bio-functions, such as the organization of ECM and hereditary connective tissue disorders. A comparison with the matrisome revealed 55 genes and highlighted *MUC16* and *FREM2*. We also contrasted the list of mutated genes with those from a transcriptomic analysis on data from Gene Expression Omnibus, with only 0.5% of the genes at the intersection of both approaches supporting the hypothesis of two different diseases that inevitably share a common genetic background but are not the same. Potential biomarkers for HSD include the five genes presented. We conclude the study by describing five potential biomarkers and by highlighting the importance of genetic/genomic approaches that, combined with clinical data, may result in an accurate diagnosis and better treatment.

## 1. Introduction

Joint hypermobility (JH) is defined as a condition in which the synovial joints move beyond the normal range of movement. Previously, when JH was associated with musculoskeletal problems, it was simply referred to as joint hypermobility syndrome (JHS). If JH occurs in multiple joints involving the limbs and the axial skeleton, it is then classified as Generalized Joint Hypermobility (GJH) [1]. The prevalence of GJH has been reported to be around 11% to 25% worldwide. However, this prevalence varies significantly between ages, genders, and ethnicities, and GJH is more common in children and young adults. The standard method of assessment for GJH is the Beighton score [2]. In clinical practice, JH alone does not necessarily lead to clearly identifiable symptoms; it is not considered a disease and, therefore, is not set for diagnosis [3]. Currently, if GJH is combined with systemic manifestations of generalized connective tissue disorder; musculoskeletal problems, such as pain, subluxation, dislocation, and premature osteoarthritis; or a positive family history, it is clinically diagnosed as hypermobile Ehlers–Danlos syndrome (hEDS) [4].

hEDS is one of the thirteen different types of Ehlers–Danlos syndrome, a heterogeneous group of hereditary disorders of connective tissue with specific ways of affecting the body. Each type is characterized by specific clinical criteria. However, the cause at the molecular level and the genetic characterization are unknown for hEDS, which is commonly confused with JHS. The most recent information (from 2021) on the updated EDS classification with the inheritance patterns, genes, and proteins associated with each EDS subtype is presented in [5]. Still, no candidate genes or proteins have been associated with hEDS.

**Figure 1:**
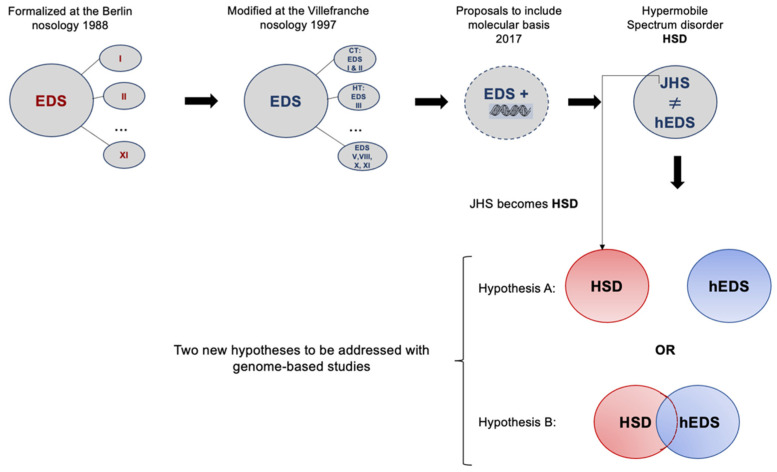
The continuous struggle to characterize joint hypermobility from 1998 to the current nosology established in 2017, in which two phenotypes are recognized. Hypothesis A identifies two diseases that have overlapping symptoms but are genetically different, while Hypothesis B presents two diseases with overlapping symptoms and common genetic backgrounds.

The origin of the formal name of EDS dates back to 1936 [6] after Edvard Ehlers (1901) and Henri-Alexandre Danlos (1908) [7] identified people with noticeable variations in the mechanical properties of their skin. It was not until 1986, after a meeting in Berlin, that a new nosology was proposed. The Berlin nosology recognized eleven subtypes numbered I to XI. More research led to the Villefranche nosology (1997) [8], which simplified the eleven classifications into six in 1997. Further clinical and molecular characterizations, as well as the lack of genetic diagnosis, raised concerns about EDS nosology again. Another EDS meeting in New York in May of 2016 led to the current nosology. The latest meeting of the International Consortium on the Ehlers–Danlos syndromes [9] revised the classification that replaced the Villefranche nosology with EDS and the Brighton criteria with JH. It introduced a new classification of hypermobility spectrum disorders (HSD), which JHS falls into [10]. It was established in Malfait’s work that JHS became HSD. Hence, it is now official—cases are classified as hEDS or HSD, and the latter is diagnosed in patients with symptomatic joint hypermobility who do not satisfy the current hEDS diagnostic criteria. Whether hEDS and HSD are the same is still not final; the overlap in clinical manifestations of HSD and hEDS requires complementing it with molecular descriptions and genomic studies to characterize both diseases further. Today, we are down to two hypotheses from the previous three presented by Castori and Colombi in 2015 [11]. Whether HSD and hEDS are entirely unrelated or whether their phenotypes share a common genetic background remains unknown (Figure 1).

HSD is a group of clinically relevant and different conditions related to joint hypermobility, whilst hEDS, characterized by JH and fewer, less severe cutaneous manifestations, tends to be more limited to musculoskeletal manifestations. Both conditions have been associated with various other conditions, such as functional gastrointestinal disorders, dysautonomia, and mast cell activation syndrome. However, what is common to them is that currently, there are no associated genes for these conditions and no accurate diagnosis leading to proper treatment. We aim to find genetic biomarkers that characterize HSD but are also able to distinguish it from hEDS. This work presents a landscape of genetic mutations through whole-exome sequencing in samples from patients clinically diagnosed with HSD (previously GJH). To contrast mutated genes in HSD to transcriptional changes in another group of patients diagnosed with hEDS/JHS (prior to 2017 nosology), we used a whole-transcriptomic case–control microarray dataset downloaded from Gene Expression Omnibus.

## 2. Materials and Methods

### 2.1. Data

DNA from blood samples of 5 patients, two of them with a familial relationship, mother (patient S2) and daughter (patient S1), were processed for whole-exome sequencing. The referring physician established the clinical diagnosis as generalized HSD by thoroughly reviewing the clinical information to ensure an accurate phenotype. In addition, all other symptoms were recorded in the patient’s clinical profile. The details of the clinical data are shown in Table 1, and the pedigrees are shown in Supplementary Figure S1.

The genomic DNA was extracted from 100 µL of whole blood from the affected individuals using the DNeasy Blood and Tissue Kit (Qiagen, Hilden, Germany). Genomic DNA was quantified using Qubit dsDNA BR Assay Kit (Invitrogen, Carlsbad, CA, USA). Quality was determined spectrophotometrically using a Nanodrop One (Thermo Fisher Scientific, Waltham MA, USA). Sequencing libraries were prepared using Library Preparation EF 2.0 with Enzymatic Fragmentation and the Twist Universal Adapter System (Twist Bioscience, San Francisco, CA, USA) according to the manufacturer’s instructions, and exome hybridization was achieved following the Twist Fast Hybridization Target Enrichment Protocol coupled with the Twist Comprehensive Exome Panel (Twist Bioscience, San Francisco, CA, USA). All libraries were quantified with the Qubit dsDNA BR Assay Kit (Invitrogen, Carlsbad, CA, USA), library size was analyzed in a QSep 400 (BiOptic, New Taipei City, Taiwan), and sequencing was performed using an SP flow cell in a NovaSeq 6000 (Illumina, San Diego CA, USA) in a 101 bp paired-end reads configuration.

The DNA sequence was mapped to the published human genome build UCSC hg38/GRCh38 reference sequence using the latest internally validated version of Burroughs Wheeler Aligner (BWA) BWA-Mem v0.7.8 [12]. The hg38 reference genome was downloaded from the UCSC Genome browser (http://hgdownload.soe.ucsc.edu/goldenPath/hg38/chromosomes/, accessed on 13 October 2021).

The whole-transcriptome Affymetrix Human Gene St 1.0 data of five hEDS/JHS human skin fibroblasts and six healthy individuals with accession number GSE77753 is publicly available in the NCBI Gene Expression Omnibus database. Raw.CEL files were pre-processed and analyzed to complement this study.

### 2.2. WES Analysis Workflow

Following a pipeline of best practices for variant calling in clinical sequencing [13], raw sequence data in FASTQ format were aligned to the reference genome sequence using BWA-Mem. A binary alignment/map (BAM) file was then created within the SAMtools package [14].

**Figure 2:**
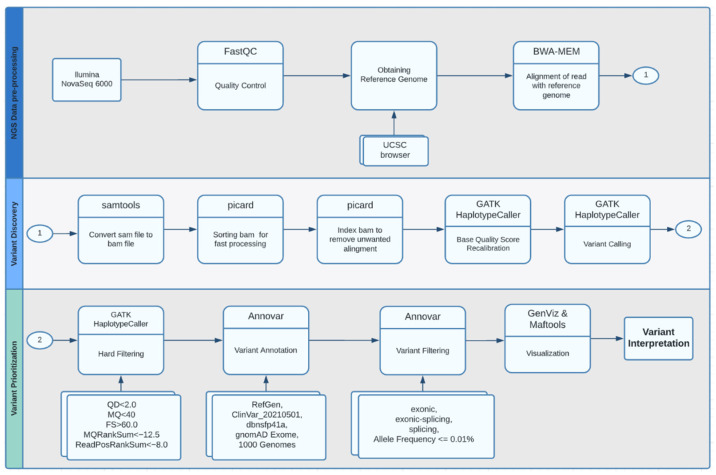
Whole-exome sequencing analysis workflow.

The workflow used in this study is based explicitly on best practices for variant calling with the Broad Institute GATK [15,16], as shown in Figure 2. This pipeline involves several steps to ensure that the alignment files are high-quality to guarantee variant calling accuracy.

#### 2.2.1. Quality Control and Pre-Processing

Quality control metrics for all *fastq* files were analyzed using FastQC [17] and filtered with trimmomatic [18] before being aligned to the reference genome. The .sam file output from the alignment was converted to a compressed .bam file, marking the PCR duplicates. The sorting and indexing of the .bam file were performed with SAMtools and Picard [19].

#### 2.2.2. Variant Calling

The GATK HaplotypeCaller [20] conducted a base-quality score recalibration and local realignment around insertion–deletion sites and regions with poor mapping quality. In addition, variant calls were identified, and complex filtering also used the GATK HaplotypeCaller.

#### 2.2.3. Variant Annotation and Visualization

Variants were annotated using ANNOVAR [21]. The RefGene database specifies known human protein-coding and non-protein-coding genes. The Clinvar_20210501 database was used to search for disease-specific variants. The dbnsfp41a database focuses on the functional prediction of variants in whole-exome data (this dataset already includes, among others, SIFT, PolyPhen2 HDIV, PolyPhen2 HVAR, MutationTaster, MutationAssessor scores), and finally, the gnomad exome and 1000 Genomes databases (1000g2015aug) were used to determine the frequency of variants in whole-exome data. Annotation files were converted to Mutation Annotation Format (MAF) files to analyze and visualize variants from large-scale sequencing studies. The analysis charts were created using the Bioconductor library Maftools [22].

### 2.3. Functional Enrichment Analysis

The functional enrichment analysis was performed using g:GOSt from g:Profiler^ß^ (version e105_e52_p16_82e8f10) with the g:SCS multiple testing correction method, applying a significance threshold of 0.05 [23] and uploading the list of genes with at least one mutation. The nomenclature of the molecular functions (MFs), biological processes (BPs), and cellular components (CCs) used the terms of the Gene Ontology Consortium [24]. In addition, the enriched canonical pathways were identified using KEGG [25], Reactome [26], and WikiPathways [27].

### 2.4. Ingenuity Pathway Analysis

The core analysis generated with the use of QIAGEN IPA (QIAGEN Inc., https://digitalinsights.qiagen.com/IPA, accessed on 6 March 2022) identified the enriched bio-functions (*p*-value < 0.01 using the right-tailed Fisher’s exact test) as well as the networks [28] using the list of genes with at least one mutation.

### 2.5. Gene Expression Profiling

Only mRNA data were downloaded from GEO Accession Number GSE77753. Samples were classified into two main groups: skin fibroblast cultures from five hEDS/JHS female patients and six unrelated healthy donors. Raw data were background-corrected using Robust Multiarray Average (RMA) [29] and normalized using Quantile Normalization [30]. Differential expression was determined using linear statistical models with arbitrary coefficients; contrasts of interest were analyzed using the Bioconductor library limma [31,32]. Correction for multiple hypotheses was applied using a false discovery rate (FDR). Genes were selected as differentially expressed on the basis of two summary statistics: a fold-change >1.68 and <7.62 in magnitude and a *p*-value < 0.09.

## 3. Results

Single-nucleotide DNA variants of the five patients diagnosed with HSD and variant-containing genes are reported. In addition, the differentially expressed genes from the dataset downloaded from GEO are also presented as their correlation or lack thereof.

### 3.1. Whole-Exome Sequencing

WES generated an average of 11 GB of sequencing data per sample. The mean coverage of the targeted regions was 140× per sample, with >98% covered with at least 30× coverage. The base-calling accuracy had, on average, a Phred quality score of 36. Variant calling on the entire genome produced ~100,000 variants per sample. Filtering out common SNPs (>10% frequency present in 1000 Genomes database) resulted in ~5000 variants per proband sample. The median number of results requiring human evaluation for each automated search ranged from 5 to 70. The total number of genes with at least one mutation (1162) is listed in (Supplementary File S1:A).

#### 3.1.1. Analysis of Variants

**Figure 3:**
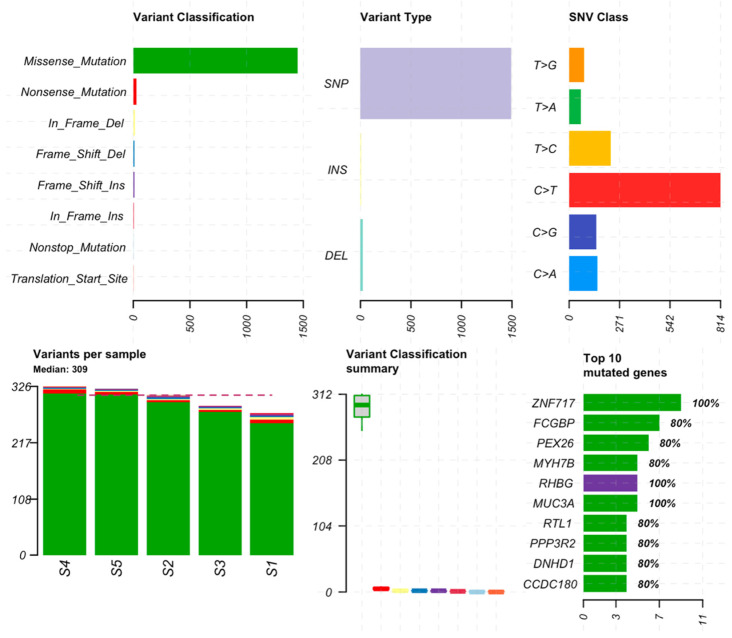
Mutation summary by variant classification, type of variant, and class (**top plots**). The number of mutations per sample, boxplots for variant classification, and the top 10 mutated genes (**bottom plots**). Color representations are green for missense mutations, red for nonsense mutations, purple for frameshift insertions, blue for frameshift deletions, yellow for in-frame deletions, dark pink for in-frame insertions, cyan for nonstop mutations and orange for translation start site. The red dotted line in the variants per sample plot indicates the median of variants for all samples.

To identify candidate disease-associated variants, databases and other metadata were used to filter putative variant calls to enhance the assessment of variants likely to impact function. Besides the usual workflow, which involves population analysis through allele frequencies and prediction of deleterious effects, we also assessed variants according to genes, transcripts, and enrichment analysis. Figure 3 presents the landscape of annotations for the genomic location and the variant class. Exonic missense, nonsense, stop-loss, frameshift, and splice-site variants are listed due to their potential effect on protein function.

**Figure 4:**
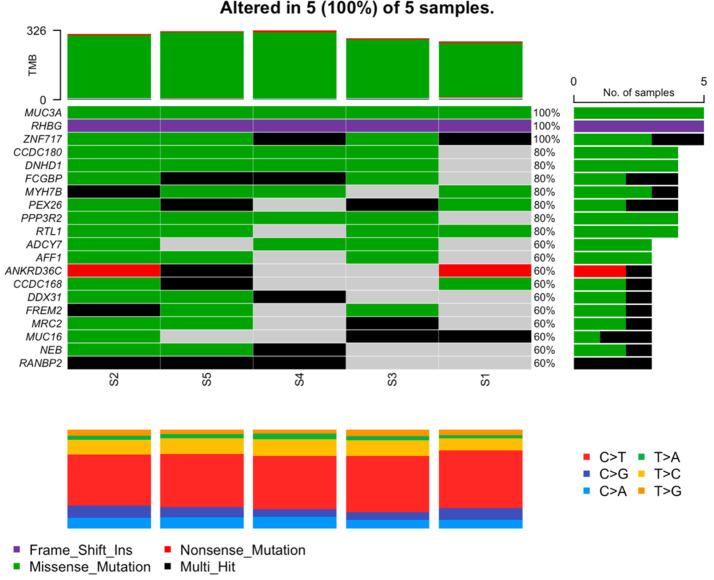
Mutation characterization of most mutated genes. Each column represents a patient; rows are genes. Here, green represents missense mutations, purple represents frameshift insertions, red represents nonsense mutations, and black represents multi-hit mutations. Gray is interpreted as no mutations found.

Missense mutations are of particular interest since they are commonly related to pathological conditions influencing susceptibility to disease or resistance to drug therapies. Significantly mutated genes according to an FDR < 0.1 along with the mutation rate, mutation type, and nucleotide changes are shown in Figure 4.

The results showed three genes that were mutated in all samples. The *MUC3A* gene with the same nonsynonymous missense mutation c.C7484T in exon 2 of chromosome 7 led to the amino acid change p.S2495L. The same frameshift insertion c.1265dupC in exon 9 of the *RHGB* gene located in chromosome 1 followed the amino acid change p.D425Rfs*18. Of the three, the *ZNF717* gene located on chromosome 3 was the only one with various mutations, all of which were nonsynonymous; see Table 2.

#### 3.1.2. Other Variants of Potential Interest

The Human Phenotype Ontology (HPO) uses the medical literature and other databases such as Orphanet, DECIPHER, and OMIM and has created 13,000 terms and over 156,000 annotations to hereditary diseases. According to the HPO database, we found a list of other mutations of potential interest using joint hypermobility as a keyword. These, along with details on our exome study, are shown in Table 3.

### 3.2. Functional Enrichment Analysis

Functional enrichment analysis identified the extracellular matrix structural constituent as the primary overrepresented molecular function (MF) based on our list of 1162 genes with at least one mutation. Similarly, the collagen-containing extracellular matrix was among the most statistically associated cellular components (CCs). On the other hand, the biological processes (BPs) identified were related to the DNA-dependent DNA replication maintenance of fidelity. The extracellular matrix (ECM)–receptor interaction was the only canonical pathway (CP) enriched and defined by KEGG. The extracellular matrix organization and degradation pathways were Reactome´s most statistically significant CPs. In addition, the resolution of D-loop Structures through Holliday Junction Intermediates was enriched (Supplementary File S1:B).

### 3.3. Ingenuity Pathway Analysis

#### 3.3.1. Core Analysis by IPA

IPA core analysis identified relevant bio-functions, such as the organization of ECM and hereditary connective tissue disorders, and DNA damage pathways. (Supplementary File S1:C). In addition, networks that included the highest number of genes with at least one mutation were those related to hereditary disorders, highlighting connective tissue disorders (Supplementary File S1:D).

#### 3.3.2. Network Analysis

**Figure 5:**
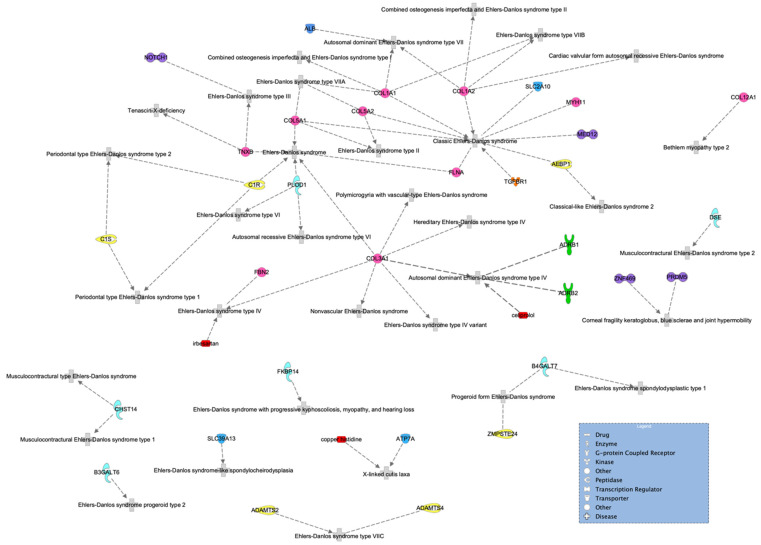
Ehlers–Danlos syndrome network of genes highly associated with the disease. Molecules are classified by shape (see legend) and colored to make them easier to follow.

**Figure 6:**
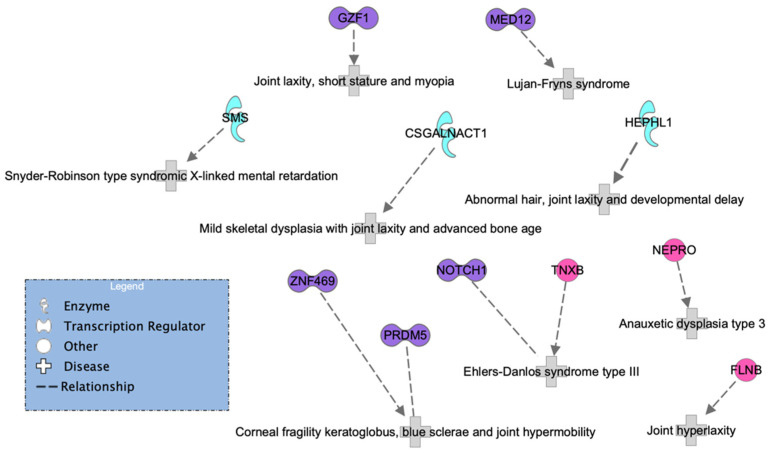
HSD network of genes known to be associated with HS according to the IPA database.

Using the IPA disease search engine, we conducted a network analysis on the genes associated with Ehlers–Danlos syndrome (Figure 5) or joint hypermobility (Figure 6).

No matches were found in a more specific search, including type III (or type 3), which is hEDS. However, we can see from the network that the transcription regulator NOTCH1, represented in purple in the top left of Figure 5, has been previously associated with hEDS. In addition, the genes *ZNF469* and *PRDM5* have been previously associated with joint hypermobility but are mainly characterized by brittle cornea syndrome.

A second and sparser network (Figure 6) was generated by only 11 proteins. Here, four out of five transcription regulators (purple) coincided in the two networks, but *MED12* was more specifically associated with Lujan–Fryns syndrome, and a new gene, *GZF1*, emerged; see Figure 6. Interestingly, a study on a Saudi family reported mutations in this gene associated with joint laxity, short stature, and severe myopia with prominent eyes. In addition, two affected members from different families exhibited multiple joint dislocations involving the elbows, hips, knees, ankles, as well as pectus carinatum and talipes equinovarus.

It is important to highlight that we observed the *FLNB* gene in the HSD network and found the *FLNA* gene in the hEDS network. It is evident that both genes are highly relevant. The *FLNA* gene encodes the protein filamin A, which participates in the development of the cytoskeleton. It also binds to integrins known to be responsible for spanning cell membranes and anchoring cells to the extracellular matrix. In addition, it has been previously associated with EDS. Similarly, the *FLNB* gene also encodes a protein called filamin B, which is involved in the development of the skeleton before birth. It is expressed in many cells and tissues in the body, including chondrocytes, a type of cell involved in cartilage formation. None of the enzymes or other genes appeared in both networks.

### 3.4. Comparison of Exome Results in HSD with Published Studies

#### 3.4.1. Comparison with the Matrisome

Functional profiling and the IPA results involving the ECM prompted us to compare our list of genes mutated in HSD vs. the matrisome, a list of more than 1000 proteins that represent the definition of the complete repertoire of ECM proteins, on the basis of homologies with known ECM proteins [33,34]. The number of shared genes between both lists was 55 (Supplementary File S2:A), and *MUC16* had the highest number of mutations, followed by *FREM2* (six and four, respectively). The functional profiling (Supplementary File S2:B) showed that both genes participated in the BPs of cell and biological adhesion as well as the CCs of the extracellular region and vesicles. Still, only *FREM2* was identified within the CP of the ECM–receptor interaction. *COL22A1* and *LAMA5* had three mutations and were involved in the MF of structural constituents of the ECM and the CP of ECM organization. However, the first one participated in the CP of collagen biosynthesis and modifying enzymes, while the latter was involved in the CP degradation of the ECM and laminin interactions (Supplementary File S2:C).

#### 3.4.2. Comparison with Differentially Expressed Genes in Skin Fibroblasts of Patients with JHS/hEDS

**Figure 7:**
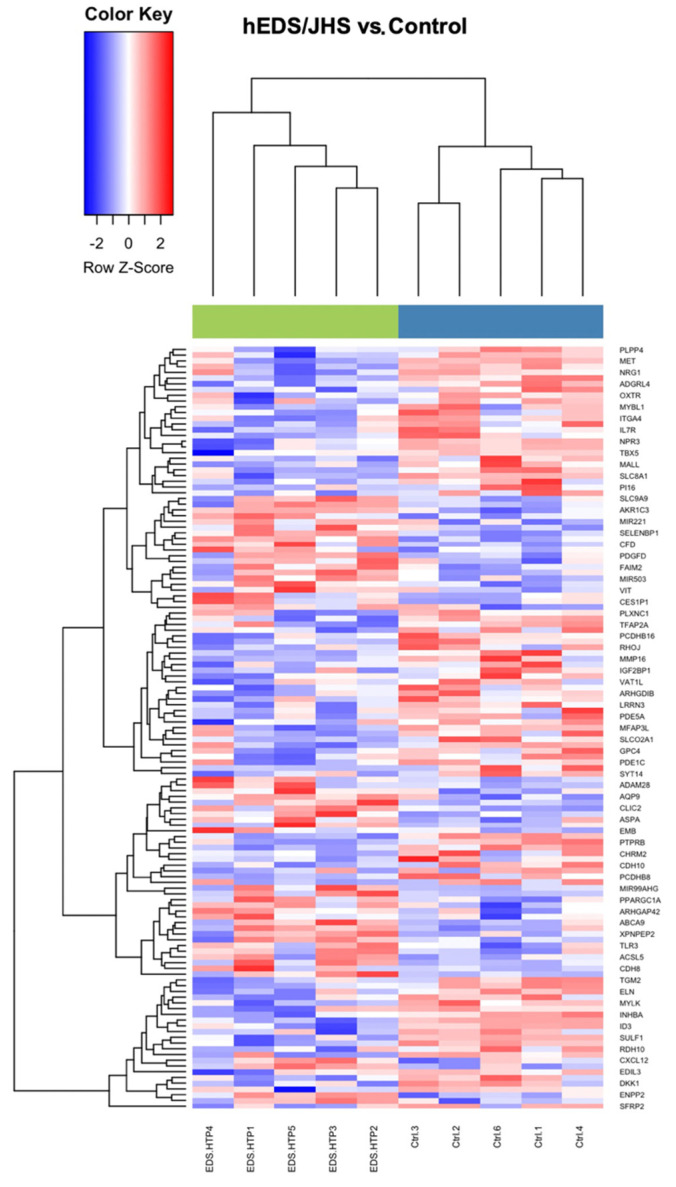
Heat map results from an unsupervised hierarchical clustering algorithm. The green bar on top represents hEDS/JHS patients and the blue bar represents the controls.

Transcriptome and proteome studies can help reveal specific biological signatures, thus providing information for the understanding of the pathomechanisms and potential biomarkers for clinical diagnosis and therapeutic interventions to inform precision medicine-based decision-making [35,36]. Data were downloaded from GEO for this analysis. They consisted of five female patients clinically diagnosed with hEDS/JHS. The differential analysis resulted in 133 differentially expressed genes (DEGs) with fold-changes ranging from 1.68 to 7.62 in magnitude and supported by *p*-values of 8.7 × 10^5^, 0.09. From this list, 83 genes (62%) were downregulated and 50 (38%) were upregulated. The pattern of expression levels between the two groups is represented in an unsupervised hierarchical cluster showing the patients in steel blue and the controls in green. The z-score color key displays upregulation in red and downregulation in blue (Figure 7).

**Figure 8:**
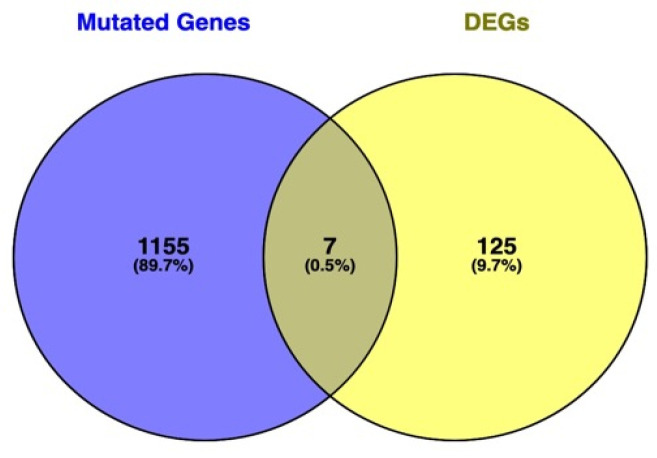
Venn diagram showing the intersection level between the list of genes found to be significant in both approaches: WES mutated genes and differentially expressed genes (DEGs). Genes at the intersection are *LIMCH1, ACSL5, FLG, DSP, EDIL3, PRUNE2*, and *ZFPM2*.

The Venn diagram in Figure 8 shows the shared genes in the two lists: the mutated genes from the whole-exome sequencing analysis in HSD and the DEGs in skin fibroblasts of patients with JHS/hEDS (Supplementary File S2:D).

#### 3.4.3. Comparison with the Proteome Profiling in Dermal Myofibroblast of Patients with hEDS

The proteome profiling of hEDS patients´ dermal myofibroblast identified the differential expression of proteins principally implicated in cytoskeleton organization, energy metabolism and redox balance, proteostasis, and intracellular trafficking [37]. Our list of 1162 genes with at least one mutation in HSD patients and these 183 differentially expressed proteins shared only five genes (*MYO1C*, *TXNDC5*, *KFT10*, *PEPD*, and *CKAP4*) (Supplementary Files S2:E).

## 4. Discussion

Despite the constant efforts for an accurate diagnosis, the struggle to DIFFERENTIATE hypermobility spectrum disorder (HSD) from the hypermobility type of Ehlers-Danlos syndrome (hEDS) persists. The approaches have been mostly based on clinical and observational parameters. However, these have proven insufficient to separate HSD from hEDS or determine if they are the same. There is a clear need for genetic and molecular characterizations of patients carefully diagnosed with either phenotype. Malfait and collaborators, in their recent publication in *Nature* (2020), argued that next-generation sequencing (NGS) has facilitated the genetic diagnosis of EDS. Nevertheless, hEDS is the only one with neither genetic nor molecular associations. A potential argument is that since hEDS is commonly confused with HDS, genetic changes in patients from either group could be averaged out. In agreement with the argument of Malfait and collaborators, we also think that a future direction of this research should include multi-omics approaches, such as NGS technologies, and new analytical strategies to identify different classes of biomarkers capable of providing a more accurate description of the disease underlying molecular mechanisms.

In this work, we present a mutation landscape of patients clinically diagnosed with generalized HDS through whole-exome sequencing. Clinical details of patients can be found in Supplementary Table S1. We present gene mutation rates and patient-specific variants. Since the latest classification in 2017, no other studies have specifically focused on separating patients with HDS from those with hEDS by clinical diagnosis. To the best of our knowledge, this is the first study of this kind using NGS technologies such as whole-exome sequencing. We found two more genomic-based studies, one on gene expression data and another also using WES with a large cohort of patients. However, both studies were conducted before 2017, so a natural mixture of HDS and hEDS is expected. We used the raw data as the expression data since it was available to download from GEO and compared the list of DEGs to our list of mutated genes to find that only 0.5% of genes were shared. The WES data from the other study are still not available, perhaps due to the fact that no publication of that data has been released yet.

Three genes appeared to be mutated across all five patients in our analysis. *MUC3A* encodes membrane-bound mucins possessing two epidermal growth factor-like domains. The gene *MUC3A* is mainly expressed in the intestine [38]; it encodes membrane-bound mucins possessing two epidermal growth factor-like domains. Previous research highlighted that *MUC3A* variants were associated with inflammatory bowel disease and that this gene also promoted the progression of colorectal cancer [39]. It was reported that up to 62% of hEDS patients suffer from irritable bowel syndrome [40], and with respect to our study, three out of five cases presented gastrointestinal symptoms. In the case of *RHBG,* this gene encodes an ammonia transporter and is expressed in distal renal epithelial cells [41]. As far as we know, HSD as an independent phenotype is not associated with renal diseases [42], but urinary biomarkers have been proposed for other types of Ehlers–Danlos syndromes. Further investigation of this gene may lead to a non-invasive diagnostic urinary biomarker. Gene *ZNF717* encodes a transcription factor that appears to play a role in osteogenic differentiation [43]. Recently, a WES study identified variants in this gene, highlighting its potential involvement in autistic spectrum disorders (ASD) in the pediatric population [44]. In this sense, ASD and HSD share several clinical manifestations in adulthood [45], including proprioceptive impairment (three out of five patients in our study) and autonomic dysfunction (four out of five in our research), highlighting the theoretical involvement of *ZNF717* as a potential etiological factor behind the association between ASD and HSD in the adult population.

In the Venn diagram, only seven genes (0.5%) fall at the intersection of mutated genes from WES and DEGs from the transcriptomics data from GEO. Since the transcriptomics data were generated prior to 2017 when the new classification was established, the samples are a mixture of hEDS and HSD. The WES data comprised only HSD samples, and therefore, a low correlation was expected. However, the results suggest that new phenotype-specific studies conducted with the current classification are needed in order to compare multi-omics data. We should consider that the results are from different samples and sources with different omics technologies. We hypothesize that the transcriptome included a mixture of HSD and hEDS samples since the study was conducted before the latest classification was established in 2017.

In conclusion, we report genes and mutations as potential biomarkers for HSD. These include *MUC3A*, *RHBG,* and *ZNF717*. Our findings support the idea of two similar diseases that inevitably share a common genetic background but are not the same. From the perspective of the current status of both diseases, we lean toward Hypothesis B, described in the diagram in Figure 1. We conclude our discussion by highlighting the importance of genetic/genomic approaches to move forward with creating complementing information that, combined with clinical parameters, will result in accurate diagnoses and better treatment.

Mutation patterns within the context of what is known about EDS and HSD indicate that genes will be found to be associated with either phenotype, different genes perhaps but with similar functions. Such is the case of *FLNA* and *FLNB*. The first one encodes an actin-binding protein, which participates in cytoskeleton formation, anchors various proteins in the cytoskeleton, and regulates cell adhesion and migration [46]. A mutation in the *FLNA* is the most common cause of periventricular nodular heterotopia. However, some patients with an *FLNA* mutation have also been shown to have Ehlers–Danlos-like collagenopathy [47]. *FLNB*, on the other hand, encodes a cytoplasmic protein that regulates the structure and activity of the cytoskeleton by cross-linking actin into three-dimensional networks. This gene is expressed in growth plate chondrocytes and the development of vertebral bodies. It has been reported that a heterozygous missense mutation in FLNB disrupts vertebral segmentation, joint formation, and skeletogenesis [48]. Furthermore, this is associated with the human skeletal disorder known as Larsen syndrome, characterized by congenital dislocations of the hip, knee, and elbow [49]. In any event, the discovery of one is necessary for the discovery of the other.

The matrisome is a list of more than 1000 proteins that represent the definition of the complete repertoire of ECM proteins (33, 34). The comparison of our list of mutated genes in patients with HSD vs. the matrisome that *MUC16* had the highest number of mutations, followed by *FREM2*. The first gene, *MUC16*, encodes a high-molecular-weight glycoprotein expressed by the human body´s epithelial cell surfaces [50]. It is a well-established serum biomarker of ovarian cancer and is a potential therapeutic target for this disease [51]. However, the signaling pathways via the MUC16 cytoplasmic domain are mainly unknown, which could partially explain the unaccountable association of pathogenic predicted variants in this gene with HSD [52]. The second gene, *FREM2*, encodes an ECM protein involved in the structural adhesion of the skin epithelium to its underlying mesenchyme [53]. Mutations in this gene are associated with Fraser syndrome, an autosomal recessive disease, secondary to the failure of the apoptosis program and the disruption of the epithelial–mesenchymal interactions during embryonic development [54]. Furthermore, *FREM2* mutations may be potential prognostic markers in colorectal cancer [55], a disease modified by *MUC3A*, a gene that was mutated in our five patients with HSD.

The transcriptome-wide expression profiling in skin fibroblasts of patients with JHS/hEDS indicated perturbation of different signaling cascades required for homeostatic regulation either during development or in adult tissues. Furthermore, altered expression of several genes involved in the maintenance of ECM architecture and homeostasis and cell–cell adhesion was also observed [56]. In this regard, we identified the ECM as a gene ontology (GO) term, canonical pathway, and bio-function enriched with genes with at least one mutation in HSD patients. This finding is relevant because ECM synthesis and remodeling regulation have been associated with heritable connective tissue disorders [57]. In addition, other GO terms, such as cell adhesion molecule binding and cytoskeleton motor activity, were overrepresented. Recently, it has been proposed that aberrations in cell adhesion and cytoskeleton dynamics could drive the abnormal properties of the connective tissue and be responsible for the pathogenesis of HSD/EDS [58]. In this sense, *DSP*, an adhesion molecule-encoding gene identified with only one mutation in our list of mutated genes in patients diagnosed with HSD became relevant. Its encoded protein, desmoplakin, is involved in forming desmosomes, a specialized cell–cell junction complex essential for maintaining tissue architecture [59]. Therefore, this gene may represent a potential biomarker of HSD according to the hypothesis of Castori that establishes that a single inherited variant may serve as a predisposing factor or “susceptibility locus”. Nevertheless, this factor results in the expression of the JHS/hEDS only when supplemented with other inherited or acquired factors [60]. Although it is worth following up on this gene, the association in our study was weak.

The comparison of our list of mutated genes in HSD vs. DEGs in skin fibroblasts of patients with JHS/hEDS [61] identified less than one percent in common. However, this finding does not necessarily mean that the genes are not somehow correlated. We should consider that the results are from different samples and sources with different omics technologies. We hypothesize that the transcriptome included a mixture of HSD and hEDS samples since the study was conducted before the latest classification was established in 2017. Furthermore, a comparison of our list vs. the proteome profiling in dermal myofibroblasts of patients with hEDS [62] identified only five genes. One of them, prolidase (peptidase D), encoded by *PEPD*, is a ubiquitously expressed cytosolic metalloproteinase essential in protein metabolism, collagen turnover, and matrix remodeling [63], representing a potential candidate to be evaluated in larger cohorts of patients with HSD. On the other hand, a key factor identified by proteomic analysis in hEDS fibroblasts was S1004A, a calcium-binding protein involved in the fibroblast-to-myofibroblast transition, supporting a proinflammatory milieu characterized by an excessive matrix metalloproteinase-mediated ECM degradation [62]. This protein is a potential target for therapeutic strategies in hEDS, highlighting the necessity to complement our whole-exome studies with a proteomic approach in fibroblasts of patients with HSD.

## Figures and Tables

**Table 1:** Clinical data of patients involved in the study. The "+" sign indicates positive for the parameter.

Phenotype	S1	S2	S3	S4	S5
Sex	F	F	M	F	M
Age	13	49	25	19	70
Family history	+	+	+	+	+
The Five-Point Questionnaire, 5PQ [1]	+	+	+	+	+
Beighton Score	6/9	6/9	7/9	7/9	6/9

**Table 2:** Mutations in genes *MUC3A, RHBG,* and *ZNF717*.

Gene	Sample	Exon	Ref	Alt	txChange	aaChange	Variant Class
*MUC3A*	All	exon 2	C	T	c.C7484T	p.S2495L	Missense_Mutation
*RHBG*	All	exon9	-	C	c.1265dup	p.D425Rfs*18	Frame_Shift_Ins
*ZNF717*	All	exon 4	T	C	c.A191G	p.Y64C	Missense_Mutation
	S1, S4	exon 5	G	C	c.C2146G	p.Q716E	Missense_Mutation
	S1, S4	exon 5	C	A	c.G1832T	p.R611I	Missense_Mutation

**Table 3:** Mutations of genes associated with joint hypermobility according to the Human Phenotype Ontology.

Sample	Ref > Alt	Genotype	Gene Function	Reference Gene	Exonic Function	avsnp150
S1	A > G	het	exonic	*DPYD*	nonsyn SNV	rs1801265
	C > T	het	exonic	*ATP6V0A2*	syn SNV	rs138886791
	C > T	het	exonic	*FBLN5*	syn SNV	rs746630839
	C > T	het	exonic	*FBN1*	nonsyn SNV	rs146726731
S2	G > A	het	exonic	*SON*	syn SNV	rs61746013
S3	- > A	het	splicing	*COL12A1*	-	
	C > T	het	exonic	*C1S*	syn SNV	rs148573885
	G > A	het	exonic	*CREBBP*	nonsyn SNV	rs772991403
	G > T	het	exonic	*SON*	nonsyn SNV	rs142482063
	A > G	het	exonic	*SON*	syn SNV	rs144018038
S4	A > G	het	exonic	*DPYD*	nonsyn SNV	rs1801265
	C > T	het	exonic	*NOTCH3*	nonsyn SNV	rs199620476
S5	C > T	het	exonic	*CREBBP*	syn SNV	rs61754523

## Data Availability

Processed data are available as part of this submission. Raw data from WES are available upon request from the corresponding authors.

## Supplementary Materials

The following supporting information can be downloaded at: https://www.mdpi.com/article/10.3390/genes13071269/s1, Figure S1: Pedigrees of four families with a proband with HSD diagnosis, Table S1: Clinical data of patients involved in the study, File S1:A. The 1162 mutated genes in HSD, File S1:B. Functional profiling HSD, File S1:C. IPA Bio-functions in HSD, File S1:D. IPA Networks in HSD; File S2:A. The 55 genes HSD vs. matrisome, File S2:B. Functional profiling HSD, File S2:C. FP Genes with >2 mutations, File S2:D. Seven genes HSD vs. hEDS JHS, File S2:E. Five genes HSD vs. proteome.
