# Supplementary material for: Genomic Characterization by Whole-Exome Sequencing of Hypermobility Spectrum Disorder
Source: Genes (Basel). 2022 Jul 18;13(7):1269. doi: 10.3390/genes13071269 (PMC9319525; doi:10.3390/genes13071269)
Supplement: Supplementary file 1 [file genes-13-01269-s001.zip › Supplementary Table S1.pdf]

Supplementary Table S1. Clinical Data of patients involved in the study

**Phenotype**	**S1**	**S2**	**S3**	**S4**	**S5**
Sex	F	F	M	F	M
Age	13	49	25	19	70
Family history	+	+	+	-	+
The Five-Point Questionnaire 5PQ [1]	+	+	+	+	+
Beighton Score	6/9	6/9	7/9	7/9	6/9
Mucocutaneous					
Unusually soft or velvety skin	+	+	+	+	+
Mild skin hyperextensibility	+	-	+	+	-
Unexplained striae	-	-	-	-	-
Bilateral piezogenic papules of the heel	-	-	+	+	-
Recurrent or multiple abdominal hernia(s)	-	+	-	-	-
Atrophic scarring	-	-	+	-	-
Pelvic floor, rectal, and/or uterine prolapse	-	-	-	-	-
Marfanoid features					
Dental crowding and high or narrow palate	-	-	-	-	+
Mitral valve prolapse	-	-	-	-	-
Arm span-to-height ratio ≥1.05	+	+	-	-	-
Arachnodactyly	-	+	-	+	-
Aortic root dilatation with Z-score >+2	-	-	-	-	-
Musculoskeletal					
Musculoskeletal pain	+	+	-	+	+
Chronic widespread pain	-	-	-	-	-
Recurrent joint dislocations	-	-	+	+	-
frank joint instability	-	+	+	+	+
Mild scoliosis	-	-	-		+
Flat foot	+	-	+	-	+
Genu o cubitus valgus	+	+	+	+	+
Gastrointestinal					
Gastroesophageal reflux	-	-	-	+	+
Defecatory dysfunction	-	-	+	+	-
Neuropsychiatric					
Impaired memory and concentration	-	-	-	+	-
Cardiovascular dysautonomia	+	-	+	+	+
Anxiety	-	-	-	+	-
Proprioception dysfunction	-	-	+	+	+
Allergies					
Rhinitis	-	-	+	-	+
Atopic dermatitis	-	-	+	+	-
Ocular					
Myopia	-	-	-	+	+
