# Supplementary figures and images for: Genomic Characterization by Whole-Exome Sequencing of Hypermobility Spectrum Disorder
Source: Genes (Basel). 2022 Jul 18;13(7):1269. doi: 10.3390/genes13071269 (PMC9319525; doi:10.3390/genes13071269)

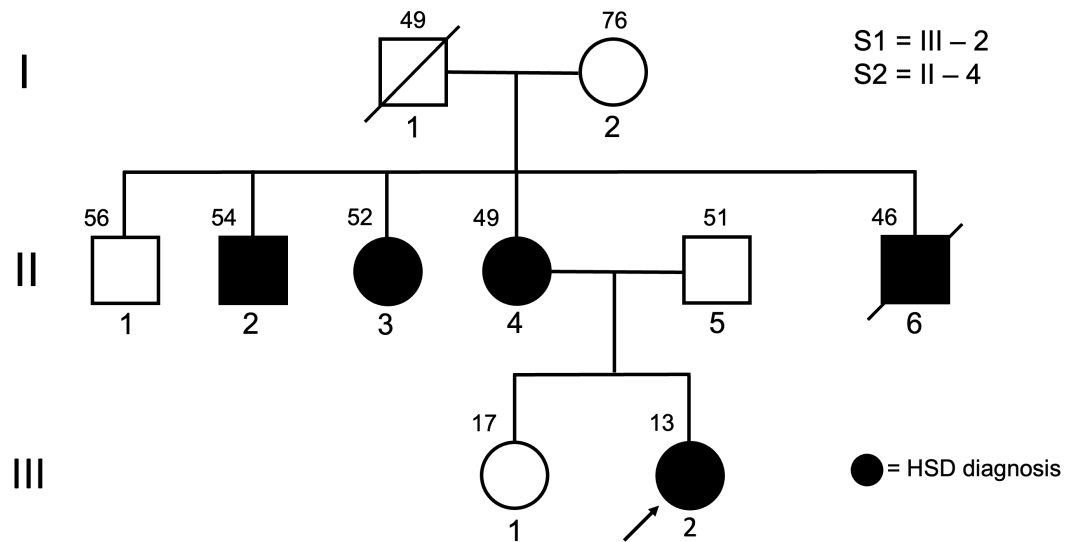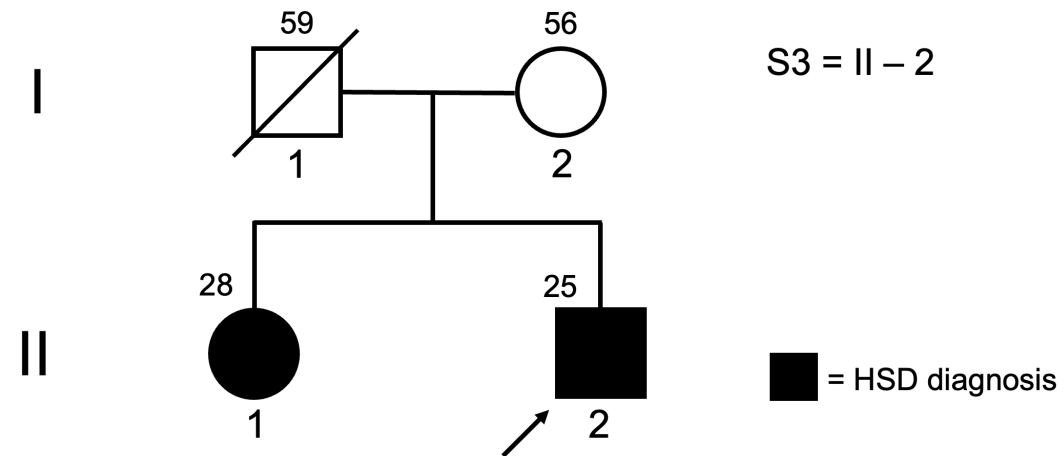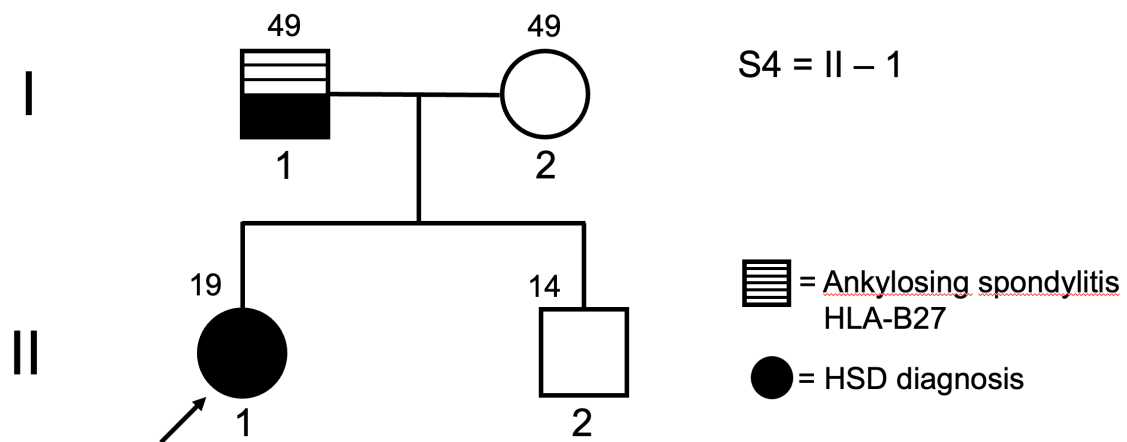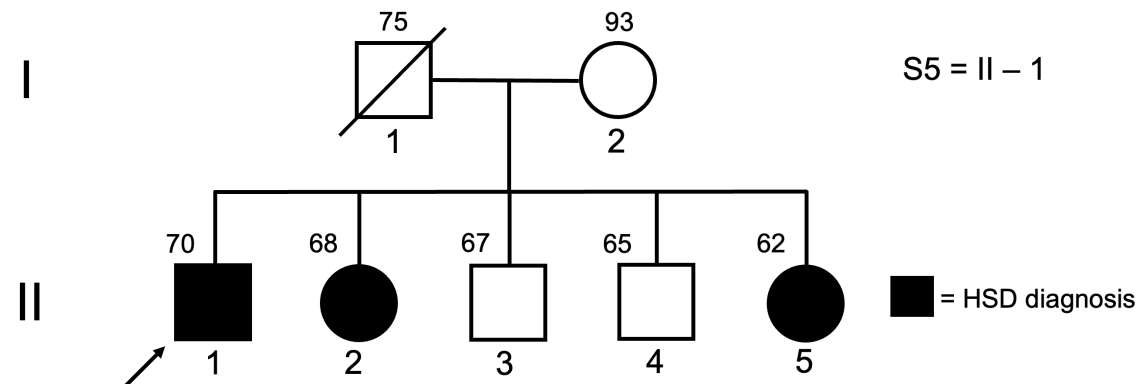

Supplement: Supplementary file 1 [file genes-13-01269-s001.zip › Supplementary Figure S1.pdf]
